# DHA Supplementation Attenuates MI-Induced LV Matrix Remodeling and Dysfunction in Mice

**DOI:** 10.1155/2020/7606938

**Published:** 2020-05-14

**Authors:** I. Habicht, G. Mohsen, L. Eichhorn, S. Frede, C. Weisheit, T. Hilbert, H. Treede, E. Güresir, O. Dewald, G. D. Duerr, M. Velten

**Affiliations:** ^1^Department of Orthopaedics and Trauma Surgery, University Hospital Bonn, Germany; ^2^Department of Anesthesiology and Intensive Care Medicine, University Hospital Bonn, Germany; ^3^Department of Cardiac Surgery, University Hospital Bonn, Germany; ^4^Department of Neurosurgery, University Hospital Bonn, Germany; ^5^Department of Cardiac Surgery, University Medical Center Oldenburg, Germany

## Abstract

**Objective:**

Myocardial ischemia and reperfusion (I/R) injury is associated with oxidative stress and inflammation, leading to scar development and malfunction. The marine omega-3 fatty acids (*ω*-3 FA), eicosapentaenoic acid (EPA), and docosahexaenoic acid (DHA) are mediating cardioprotection and improving clinical outcomes in patients with heart disease. Therefore, we tested the hypothesis that docosahexaenoic acid (DHA) supplementation prior to LAD occlusion-induced myocardial injury (MI) confers cardioprotection in mice.

**Methods:**

C57BL/6N mice were placed on DHA or control diets (CD) beginning 7 d prior to 60 min LAD occlusion-induced MI or sham surgery. The expression of inflammatory mediators was measured via RT-qPCR. Besides FACS analysis for macrophage quantification and subtype evaluation, macrophage accumulation as well as collagen deposition was quantified in histological sections. Cardiac function was assessed using a pressure-volume catheter for up to 14 d.

**Results:**

DHA supplementation significantly attenuated the induction of peroxisome proliferator-activated receptor-*α* (PPAR-*α*) (2.3 ± 0.4 CD vs. 1.4 ± 0.3 DHA) after LAD occlusion. Furthermore, TNF-*α* (4.0 ± 0.6 CD vs. 1.5 ± 0.2 DHA), IL-1*β* (60.7 ± 7.0 CD vs. 11.6 ± 1.9 DHA), and IL-10 (223.8 ± 62.1 CD vs. 135.5 ± 38.5 DHA) mRNA expression increase was diminished in DHA-supplemented mice after 72 h reperfusion. These changes were accompanied by a less prominent switch in *α*/*β* myosin heavy chain isoforms. Chemokine mRNA expression was stronger initiated (CCL2 6 h: 32.8 ± 11.5 CD vs. 78.8 ± 13.6 DHA) but terminated earlier (CCL2 72 h: 39.5 ± 7.8 CD vs. 8.2 ± 1.9 DHA; CCL3 72 h: 794.3 ± 270.9 CD vs. 258.2 ± 57.8 DHA) in DHA supplementation compared to CD mice after LAD occlusion. Correspondingly, DHA supplementation was associated with a stronger increase of predominantly alternatively activated Ly6C-positive macrophage phenotype, being associated with less collagen deposition and better LV function (EF 14 d: 17.6 ± 2.6 CD vs. 31.4 ± 1.5 DHA).

**Conclusion:**

Our data indicate that DHA supplementation mediates cardioprotection from MI via modulation of the inflammatory response with timely and attenuated remodeling. DHA seems to attenuate MI-induced cardiomyocyte injury partly by transient PPAR-*α* downregulation, diminishing the need for antioxidant mechanisms including mitochondrial function, or *α*- to *β*-MHC isoform switch.

## 1. Introduction

Coronary heart disease (CHD) is a significant health concern in the western world with increasing prevalence and the leading cause of death in Europe, accounting for €60 billion in health care costs annually [1]. Timely coronary reperfusion using either percutaneous coronary intervention (PCI) or thrombolytic therapy is the most effective strategy for limiting infarct size, preserving left-ventricular (LV) function, and therefore preventing myocardial injury and the development of heart failure [2]. Despite early onset therapies, in hospital mortality has risen up to 14% and productivity loss accounts for 38% of CHD-related health care costs [1]. Therefore, novel therapeutic approaches are required to reduce MI size, preserve LV function, and improve clinical outcomes after MI.

Although an early intervention reestablishing coronary perfusion is essential for myocardial salvage after MI, reperfusion itself triggers a further injury [3]. This so-called ischemia/reperfusion (I/R) injury is an inherent response to the restoration of blood flow involving numerous mechanisms including the increased generation of reactive oxygen species (ROS), acute calcium overload of cardiomyocytes, and opening of the mitochondrial permeability transition pore (MPTP), leading to uncoupled oxidative phosphorylation and thus contractile dysfunction. These insults further aggravate myocardial remodeling after MI, through increased generation of proinflammatory and proapoptotic molecules resulting in myocyte death, collagen deposition, and scar formation, exacerbating the development of heart failure [4, 5]. In summary, reperfusion itself induces additional cardiac damage that is responsible for up to 50% of infarct size, making this cascade of events a viable target for therapeutic interventions [3]. Currently, there is no effective clinical therapy preventing the deleterious consequences of myocardial I/R injury. Therefore, attenuating I/R injury is an important target for cardioprotection and a promising therapeutic approach to improve clinical outcomes after an acute MI.

Inflammation is pivotal for the development of heart failure, and an unrestricted inflammatory response is associated with worse prognosis after MI [6, 7]. Furthermore, excessive increases in inflammatory mediators, e.g., cytokines, have been shown to induce myocardial injury, including impaired cardiomyocyte contractility and excessive myocardial remodeling [8–11]. Myocardial reperfusion after ischemia generates an imbalance between reactive oxygen species (ROS) and the capacity of cells to defend against them, leading to increased ROS generation. The inducible transcription factor peroxisome proliferator-activated receptor-*γ* (PPAR-*γ*) regulates various cardiovascular processes and reduces I/R injury-induced cardiac inflammation and ROS generation [12]. Elevated ROS levels consume and surpass the antioxidant capacity of the injured myocardium, significantly contributing to oxidative stress generation and affecting protein function, resulting in myocardial damage with morphological and functional abnormalities [13]. In cardiac I/R injury, significant sources of ROS are inflammation-induced phagocyte-type NAD (P) H oxidase and mitochondrial metabolism-associated fatty acid oxidation [14, 15]. Excessive ROS generation opens the mitochondrial permeability transition pore (MPTP) further contributing to myocardial injury and contractile dysfunction. Mitochondrial uncoupling proteins (UCP) have been shown to protect cardiomyocytes from ROS-induced cell death and heart failure. Furthermore, UCP overexpression has been reported as an adaptive mechanism against oxidative stress in various cardiac pathologies. ROS overproduction and oxidative stress play key roles for the development of cardiac injury, promoting complications of cardiac reperfusion [16]. Thus, preventing I/R injury-induced ROS generation is an important target for the development of novel strategies to preserve cardiac function after MI.

Polyunsaturated fatty acids (PUFAs) are a group of metabolic active lipid molecules. The marine omega-3 fatty acids (*ω*-3 FA), eicosapentaenoic acid (EPA), and docosahexaenoic acid (DHA) are beneficial for health outcomes [17], mediating cardiovascular benefits in preclinical studies and improve clinical outcomes in patients with MI, potentially through modulation of inflammation and antioxidant effects [18, 19]. Long-lasting *ω*-3 FA intake is reported to reduce mortality up to 45% and morbidity (myocardial infarction, arrhythmia) in adult patients with cardiovascular disease [20]. Consequently, supplementation is recommended for patients with prevalent CHD such as a recent MI [21, 22]. DHA is either obtained from diet but can also be synthesized from EPA [23, 24]. Thus, various investigations used combinations of DHA and EPA to investigate *ω*-3 FA effects on cardiac health. The OMEGA-REMODEL trial proved that high-dose DHA/EPA supplementation beginning at the onset of MI improved LV function after 6 months [18]. However, underlying mechanisms, signaling pathways, and effectors of DHA/EPA-mediated prevention of I/R injury-induced cardiac dysfunction in acute MI remain to be revealed [24, 25]. DHA/EPA modulate numerous receptors and decrease the generation of intracellular reactive oxygen species with a subsequent diminished activation of redox-sensitive transcription factors through its incorporation into cellular membranes. In addition to inflammatory resolution, DHA has numerous effects that include oxygen consumption, mitochondrial energy metabolism, contractile function, calcium signaling, and ROS generation, potentially protecting the cardiovascular system [26–28].

Previous attempts to translate cardioprotective strategies for I/R injury from the experimental into the clinical setting have not been successful, potentially due to an incomplete understanding of the underlying molecular mechanisms including inflammation, oxidative stress, calcium overload, mitochondrial dysfunction, and different cell types affected [29]. However, most approaches targeted just one mechanism, but several of these mechanisms interact. Therefore, understanding these interactions and targeting multiple mechanisms are essential to prevent I/R injury-induced cardiac dysfunction. DHA and EPA interact with many mechanisms that are associated with the development of I/R injury-induced cardiac dysfunction including inflammation, ROS generation, matrix remodeling, and mitochondrial metabolism [26]. Therefore, *ω*-3 FA are promising therapeutics and understanding its interactions in myocardial I/R injury may help to reduce infarct size, prevent the development of LV dysfunction, and improve clinical outcomes after MI.

## 2. Materials and Methods

### 2.1. Animal Protocol

All animals were handled according to the animal protocol and to the EU Directive 2010/63/EU for animal research. Experimental procedures have been approved by the government animal care and use committee “Landesamt für Natur, Umwelt und Verbraucherschutz NRW” (50.203.2-BN 43, 28/01). 20–25 g and 10–12 weeks old male C57BL/6 mice were purchased from Charles River (Sulzfeld, Germany). To limit transportation and social stress, mice were housed at our facility for at least 7 days prior to the experiments. Animals were placed in plastic cages filled with autoclaved bedding in a filtered flow cage rack on a 12-hour light/dark cycle with free access to water and standard rodent chow. All mice were sacrificed by cervical dislocation at the end of the experiment.

### 2.2. Closed Chest Mouse Model of LAD Occlusion

Before surgery perioperative analgesia was performed using carprofen 5 mg/kg s.c. and Temgesic 0.1 mg/kg s.c. Anesthesia was then induced with 3% isoflurane (Forene®, Abbott) and maintained with 0.8% isoflurane in 100% O_2_. Left parasternal thoracotomy was performed for implantation of the ligature (8-0 Prolene suture, Ethicon, Norderstedt, Germany) around the left descending coronary artery (LAD). The suture ends were threaded through a sterile PE10 tube (Becton Dickinson, Franklin Lakes, NJ, USA), exteriorized through the thoracic wall, and stored subcutaneously [30]. After chest closure, cefuroxime suspension was injected i.p. (50 mg/kg, Zinacef; Bristol-Myers Squibb, Munich, Germany) for antibiotic prophylaxis. Mice were allowed to recover for 7–10 days from initial surgery intervention.

### 2.3. Induction of Myocardial Ischemia and Reperfusion (I/R)

Myocardial ischemia was induced under the same analgesic and anesthetic measures as described above. The LAD ligature ends were connected to heavy metal picks, and LAD occlusion for 60 min was achieved by pulling the picks apart as described previously [30]. Myocardial ischemia was confirmed by visualization of ST segment elevation in EKG lead II of Einthoven. The hearts were reperfused after removal of the LAD ligature. Persistence of ST-segment elevation confirmed myocardial infarction. After reperfusion, the hearts were excised at different time points, dissected free from the atria and great vessels, and rinsed in ice-cold cardioplegic solution.

### 2.4. Experimental Groups and Protocols

To evaluate whether *ω*-3 FA pretreatment associated mechanisms mediating cardioprotection for I/R injury-induced LV dysfunction, after implantation of the LAD ligature, C57BL/6N mice were randomly distributed to receive either DHA or control diet (CD) beginning 7 days prior to I/R and for the duration of the experiment. DHA and control diets were identical with the exception of the composition of the *ω*-3 FA, and 7 days of diet supplementation has shown to increase DHA concentrations and attenuate inflammation and oxidation in other pathologies [31–33]. Linoleic acid was used as a source for *ω*-6 FA, and amounts were similar in CD and DHA diets. However, total *ω*-3 FA contents were similar in control and DHA diets, but 37% *ω*-3 FA content in the DHA diet was DHA and the remaining half was linolenic acid from flaxseed oil, while in the control diet, the entire amount of *ω*-3 FA was linolenic acid (Table 1).

A purified diet with higher linolenic acid concentrations that is metabolized to arachidonic acid was chosen to isolate the effects of preformed DHA supplementation from those of the precursor *α*-linolenic acid and to avoid variability in standard chows [34].

#### 2.4.1. In Vivo Functional Analysis Using Millar® Pressure-Volume Left Heart Catheter

Anesthetized mice (0.8% isoflurane) were ventilated, and the jugular vein was cannulated with microenathane-033 tubing for hypertonic saline administration. After warming, the conductance catheter probe was advanced into the LV through the right carotid artery. Data collection was initiated after baseline stabilization. The Millar catheter uses conductance to determine relative volume units or “RVU.” Once RVU's are measured, we then use a known volume of blood from the individual mouse using mock-up cylinders with known volumes to convert to a known volume [35]. Furthermore, inferior vena cava occlusion was used to measure the end-systolic pressure-volume relationship (ESPVR) and end-diastolic pressure-volume relationship (EDPVR), which are the index of ventricular filling pressures.

#### 2.4.2. Immunohistochemistry

For immunohistochemistry, Vectastain Elite ABC kits and diaminobenzidine (AXXORA, Lörrach, Germany) were used. Cell density was described as cells/mm^2^, as previously reported and evaluated by a histological technician blinded for group assignment [31–33]. For mouse-derived antibodies, the mouse-on-mouse (M.O.M) immunodetection kit (AXXORA) was used. MAC-2 rat anti-mouse antibody (clone 3/38) was used for macrophages (Cedarlane, Ontario, Canada).

#### 2.4.3. Collagen Content

Excised hearts where fixated in 10% buffered zinc-formalin followed by paraffin embedding. 5 *μ*m sections from the level of the papillary muscle insertion were stained with hematoxylin and eosin (HE) or picrosirius red (SR), as previously described [36]. Quantitative analysis was accomplished by light microscopy with a video-image analyzer. Planimetric evaluation of collagen was performed on one section, including all four sites of the left ventricular myocardium, each at 100x magnification. Data was given as a percentage of the total left ventricular area. Collagen-stained vessels and pericardium were excluded.

#### 2.4.4. Flow Cytometry Analyses (FACS)

LV tissue was homogenized, inflammatory cells were labeled with fluorescence AB, and single-cell suspensions from the heart were generated as previously described [37]. The following antibodies from Thermo Fisher and BioLegend (San Diego, CA, USA) were used: CD45 (AFS98), F4/80 (BM-8), Gr1 (RB6-8C5), CD11c (N418), CD4 (RM4-5), CD8 (53-6.4), and B220 (RA3-6B2). In addition, the Annexin V-FITC Apoptosis Detection Kit (Thermo Fisher) was used according to the manufacturer's protocol. We performed flow cytometry on a FACS-Canto II, LSR II, and Fortessa (BD Biosciences, Heidelberg, D), and data was analyzed with FlowJo software (TreeStar, Ashland, OR, USA).

#### 2.4.5. Gene Expression Analysis

Gene expression was measured on a transcriptional level using Taqman® real-time quantitative RT-qPCR. FAM-TAMRA-linked customized primers were used in an ABI Prism 7900HT Sequence Detection System and SDS2.4 software (Applied Biosystems/Life Technologies, Karlsruhe, Germany). The mRNA expression was related to shams and GAPDH using the comparative *ΔΔ*Ct-method [38].

#### 2.4.6. Statistical Analysis

Normal distribution was tested, and data was presented as mean ± SEM. Statistical analysis was performed by two-way ANOVA and Bonferroni post hoc testing (PRISM 5.1; GraphPad, La Jolla, CA, USA). *P* < 0.05 was considered statistically significant.

## 3. Results

### 3.1. DHA Supplementation Attenuates MI-Induced Systolic and Diastolic Dysfunction

**Figure 1:**
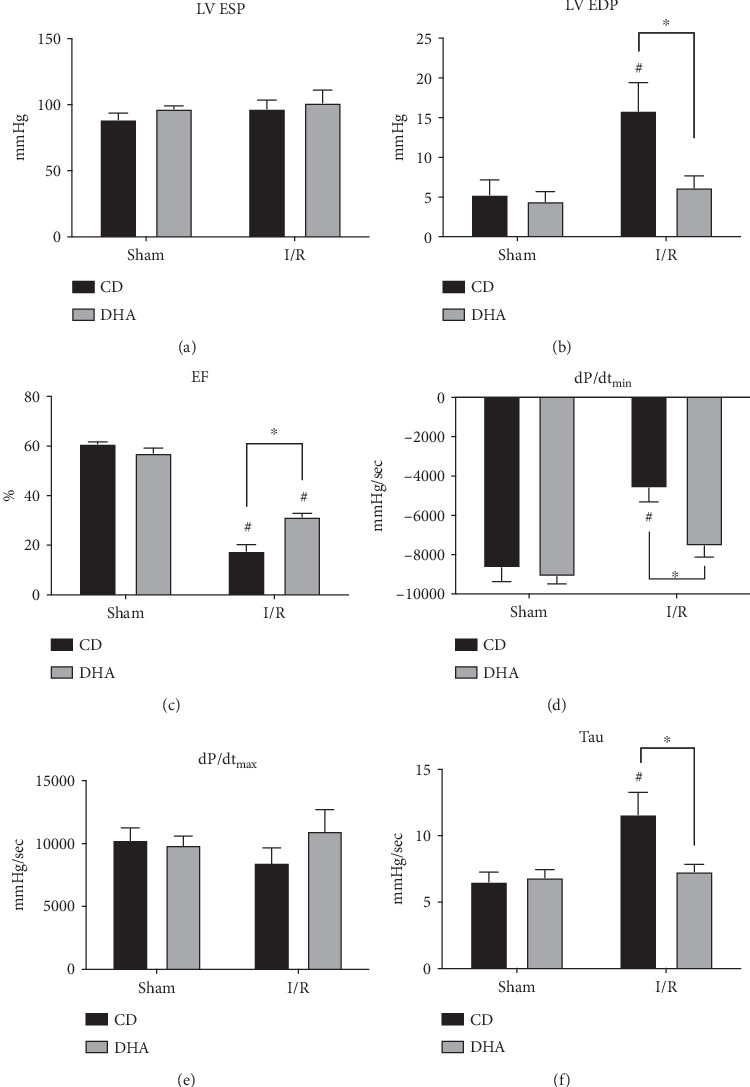
Improved cardiac function in DHA-supplemented mice after MI: functional parameters of (a) left ventricular end-systolic pressure (LVESP), (b) left ventricular end-diastolic pressure (LVEDP), (c) ejection fraction (EF), (d) peak pressure decline (dP/dt_min_), (e) peak pressure rise (dP/dt_max_), and (f) isovolumic relaxation constant (Tau), were analyzed 14 d after sham or MI in CD- or DHA-supplemented mice. *n* = 8 mice per group ^∗^*P* < 0.05.

To evaluate whether DHA pretreatment attenuates the development of MI-induced LV dysfunction, we examined the pressure-volume parameters of LV function in CD- and DHA-pretreated mice 14 days after 60 min of LAD occlusion-induced myocardial infarction (MI). Left ventricular end-systolic pressure (LVESP) was not different between sham or MI groups, regardless of DHA or CD pretreatment (Figure 1(a)). However, left ventricular end-diastolic pressure (LVEDP) significantly increased in CD mice 14 days after MI, compared to respective sham. Nevertheless, LVEDP remained at sham levels in DHA-supplemented mice and was significantly lower compared to CD mice 14 days after MI (Figure 1(b)). Mice with surgical MI had poorer ejection fraction (EF), but DHA pretreatment improved EF in the MI group compared to CD (Figure 1(c)). Peak pressure decline (dP/dt_min_) was reduced in CD-fed mice compared to sham 14 days after MI. However, dP/dt_min_ was sustained at sham levels in DHA-supplemented mice 14 days after MI and was significantly higher compared to CD mice at the same time point (Figure 1(d)). No differences were observed in peak pressure rise (dP/dt_max_) 14 days after MI in CD- or DHA-supplemented mice compared to respective sham (Figure 1(e)). Isovolumic relaxation constant (Tau) increased in CD mice compared to sham 14 days after MI. However, Tau remained at sham levels in DHA-supplemented mice and was significantly lower compared to CD mice 14 days after MI (Figure 1(f)). Two-way ANOVA indicated effects of DHA supplementation, MI, and an interaction between EF and Tau. Furthermore, statistical analyses indicated independent effects of DHA supplementation and MI on LVEDP and dP/dt_min_.

### 3.2. DHA Supplementation Modulates Cytokine Expression after MI

**Figure 2:**
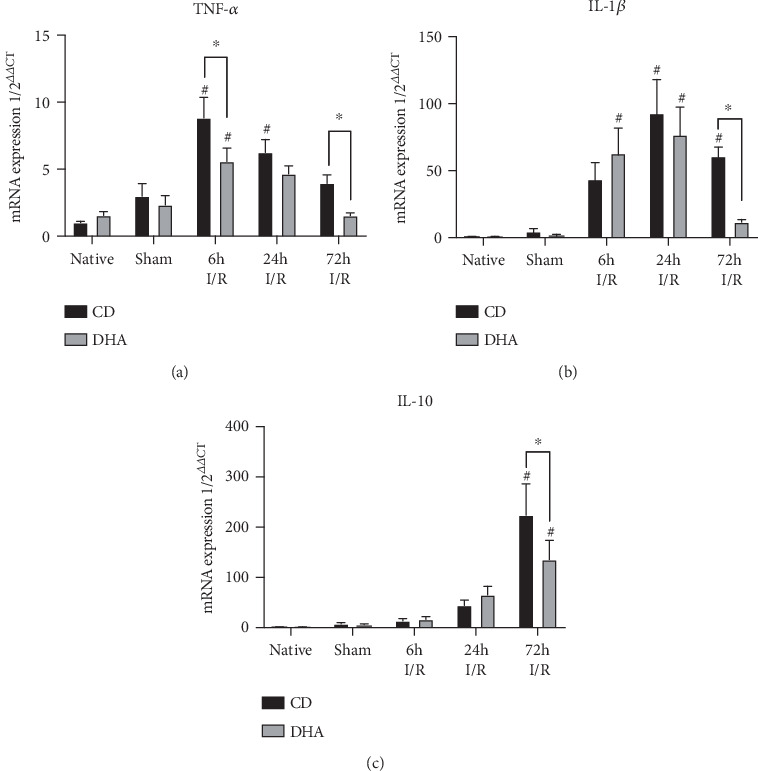
mRNA expression profile of inflammatory mediators. (a) TNF-*α*, (b) IL-1*β*, and (c) IL-10 mRNA expressions were analyzed in the murine myocardium of native, sham, and MI-exposed mice that were CD or DHA supplemented. *n* = 7 mice per group, ^∗^*P* < 0.05.

Sham procedure did not induce a sustained cytokine expression 7 days after LAD ligature implantation compared to native animals. However, the murine myocardium exhibited a marked mRNA upregulation of inflammatory cytokines after 60 min LAD occlusion compared to sham mice. TNF-*α* mRNA expression was increased in CD mice 6 h and 24 h after MI, while DHA-supplemented mice exhibited just a brief induction 6 h after MI compared to respective sham. Furthermore, MI-induced TNF-*α* mRNA expression increase was significantly lower in DHA-supplemented mice 6 h and 72 h after MI compared to CD groups (Figure 2(a)). Furthermore, IL-1*β* mRNA expression was increased in CD mice 24 h and 72 h after MI compared to respective sham, while MI-induced IL-1*β* mRNA expression occurred in DHA-supplemented mice already at 6 h and was terminated after 24 h. Furthermore, IL-1*β* mRNA expression was significantly greater in CD mice 72 h after MI compared to DHA-supplemented mice (Figure 2(b)). IL-10 mRNA was upregulated 72 h after MI in both CD- and DHA-supplemented mice. However, in mice with surgical MI, the DHA pretreatment was associated with a lower IL-10 expression than CD 72 h after MI (Figure 2(c)). MI-induced cytokine mRNA expression demonstrated a similar pattern in DHA-supplemented mice; however, induction occurred earlier, levels were lower, and increase was terminated sooner compared to respective CD mice (Figures 2(a)–2(c)). Two-way ANOVA indicated independent effects of DHA supplementation and MI on TNF-*α* mRNA expression and an effect of MI on IL-1*β* and IL-10 expressions.

### 3.3. DHA Supplementation Modifies Inflammatory Response after MI

**Figure 3:**
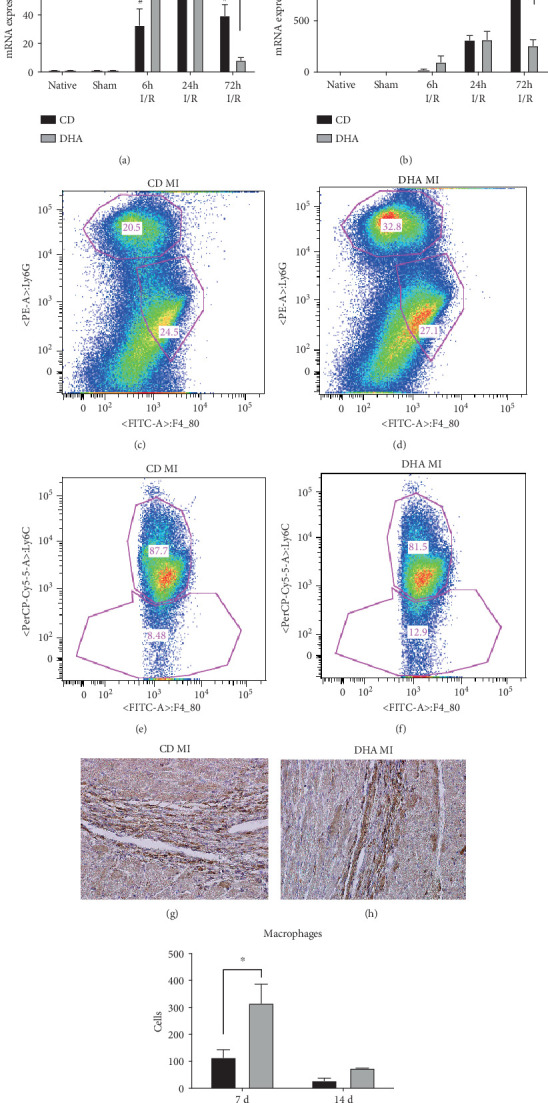
In mice with surgical MI, the DHA pretreatment was associated with a different inflammation pattern compared to the CD group. Cardiac (a) CCL2 and (b) CCL3 chemokine mRNA expressions were analyzed in the murine myocardium of native, sham, and MI-exposed mice that were CD or DHA supplemented. FACS analyses of cardiac neutrophil and macrophage populations in (c) CD or (d) DHA and phenotyping of macrophages in (e) CD- or (f) DHA-supplemented mice 3 d after MI. MAC-2 staining of representative histological left ventricular sections of cardiac macrophages in (g) CD- or (h) DHA-supplemented mice 7 d after MI and quantification (i) *n* = 7 mice per group, ^∗^*P* < 0.05.

Data shown in Figure 3 demonstrate an effect of DHA supplementation on MI-induced inflammation. CCL2 chemokine expression was increased in CD mice 6 h, 24 h, and 72 h after MI, while DHA-supplemented mice only exhibited CCL2 mRNA increases at 6 h and 24 h after MI compared to respective sham. However, CCL2 mRNA induction was significantly greater in DHA-supplemented mice 6 h after MI but terminated earlier compared to CD animals (Figure 3(a)). Furthermore, CCL3 mRNA expression was increased in CD mice compared to respective sham and DHA-supplemented mice 72 h after MI, while remained unchanged in DHA-supplemented mice compared to sham (Figure 3(b)). Fluorescence-activated cell sorting (FACS) analysis revealed greater increases in cardiac neutrophil and macrophage populations in DHA-supplemented mice compared to the CD group 3 d after MI (Figures 3(c) and 3(d)). Most importantly, cardiac macrophage population shifted from a more proinflammatory Ly6C^+^ to a more anti-inflammatory and proremodeling Ly6C^−^ phenotype in DHA supplemented compared to the CD group (Figures 3(e) and 3(f)).

In accordance with FACS analyses, MAC-2-stained histological sections revealed a large macrophage accumulation 7 days after I/R injury in both CD- and DHA-supplemented groups. However, DHA-supplemented mice showed a significantly greater infiltration of MAC-2-positive cells compared to CD-fed mice 7 days after I/R injury (Figures 3(g)–3(i)). Two-way ANOVA revealed effects of MI and an interaction between DHA supplementation and LAD occlusion on CCL2 and CCL3 mRNA expressions.

### 3.4. DHA Supplementation Modifies Collagen Expression and Reduces LV Scar Formation after MI

**Figure 4:**
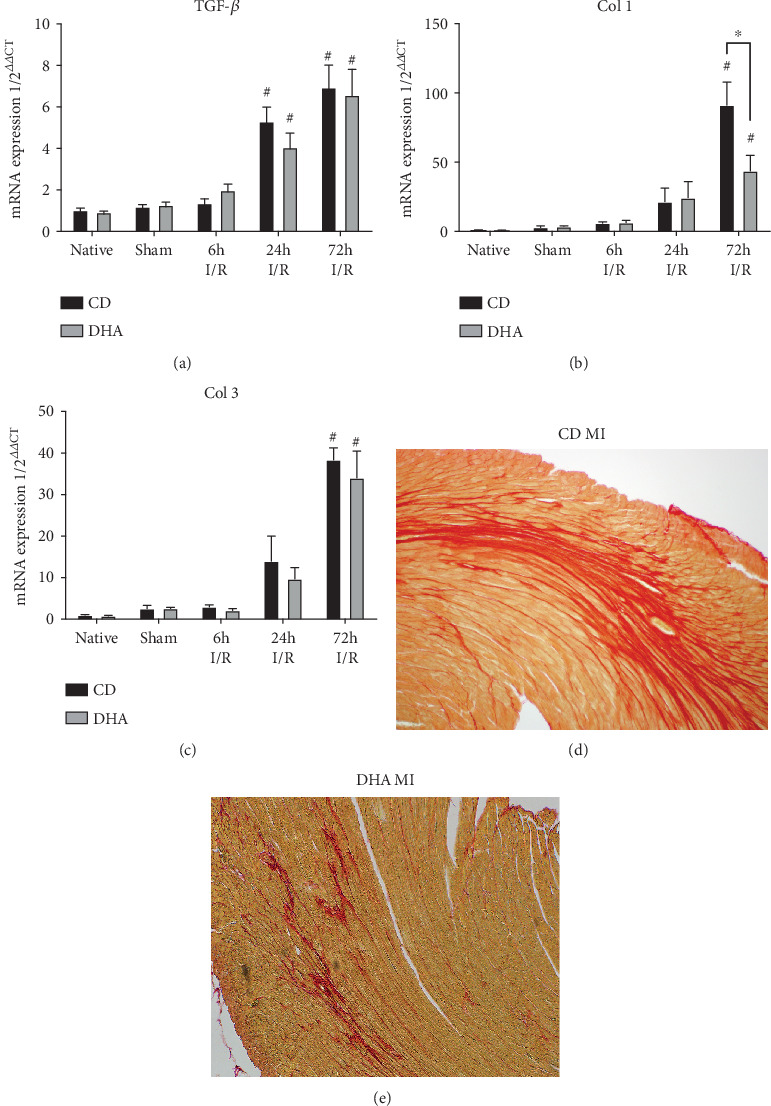
DHA supplementation modifies MI-induced myocardial remodeling: (a) TGF-*β*, (b) collagen I, and (c) collagen III mRNA expressions were analyzed in the murine myocardium of native, sham, and MI-exposed mice that were CD or DHA supplemented. *n* = 7 mice per group, ^∗^*P* < 0.05. Representative picrosirius red-stained histological sections from (d) CD- and (e) DHA-supplemented mice 14 d after I/R.

TGF-*β* mRNA was increased in both CD- and DHA-supplemented groups 24 h and 72 h after MI (Figure 4(a)). Collagen I and III mRNA expressions were also increased in both groups 72 h after MI compared to respective sham. However, collagen I mRNA expression was significantly lower in DHA-supplemented mice compared to the CD group (Figures 4(b) and 4(c)). Furthermore, analyses of picrosirius red-stained histological sections revealed less collagen deposition in DHA supplemented compared to CD mice 14 d after MI (Figures 4(d) and 4(e)). Two-way ANOVA indicated the effects of MI on TGF-*β*, collagen I, and collagen III mRNA expressions and an interaction of MI with DHA supplementation on collagen I expression.

### 3.5. Myocardial Adaptation Mechanisms toward Ischemic Injury in DHA-Supplemented Mice

**Figure 5:**
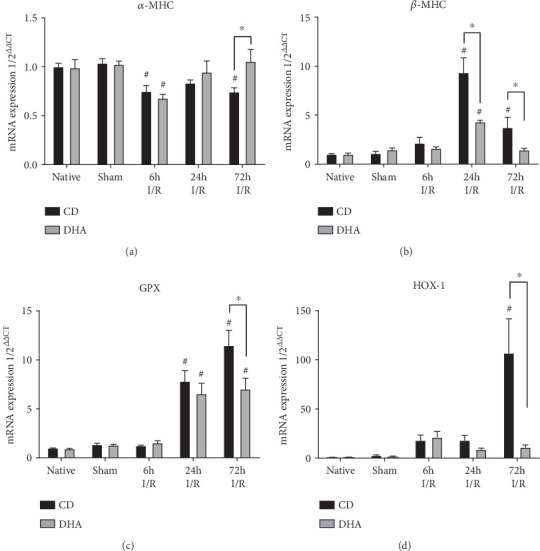
DHA-related cardiomyocyte adaptation after MI. MHC switch and induction of oxidative enzymes: myocardial (a) *α*-MHC, (b) *β*-MHC, (c) GPX, and (d) HOX mRNA expressions were analyzed in the murine myocardium of native, sham, and MI-exposed mice that were CD or DHA supplemented. *n* = 7 mice per group, ^∗^*P* < 0.05.

*α*-Myosin heavy chain (MHC) mRNA expressions were decreased in both CD- and DHA-supplemented mice 6 h after MI. However, *α*-MHC mRNA expression returned to sham levels in DHA-supplemented mice, while remained lower in CD mice compared to DHA supplemented and respective sham 72 h after MI (Figure 5(a)). Furthermore, *β*-MHC expression was significantly increased in CD mice 24 h and 72 h after I/R injury, while DHA-supplemented mice only exhibited a transient increase in *β*-MHC expression 24 h after MI compared to respective sham. Most notably, *β*-MHC expression was significantly greater in CD compared to DHA-supplemented mice 24 h after MI (Figure 5(b)). Glutathione peroxidase 1 (GPx1) mRNA expressions were increased in both CD- and DHA-supplemented mice 24 h and 72 h after MI. However, GPx1 mRNA induction was also significantly greater in CD compared to DHA-supplemented mice 72 h after MI (Figure 5(c)). Heme oxygenase 1 (HOX-1) mRNA expression increased in CD mice 72 h after MI compared to DHA supplemented and respective sham. Furthermore, HOX-1 remained at sham levels in DHA-supplemented mice. Two-way ANOVA indicated the effects of MI on *α*-MHC, *β*-MHC, GPx1, and HMOX-1 mRNA expressions. However, there were independent effects of DHA supplementation on *β*-MHC and HMOX-1 and also an interaction of MI and DHA supplementation on *β*-MHC, GPx1, and HMOX-1 mRNA expressions.

### 3.6. DHA Preconditioning Impacts mRNA Expression of Enzymes Involved in Fatty Acid Metabolism

**Figure 6:**
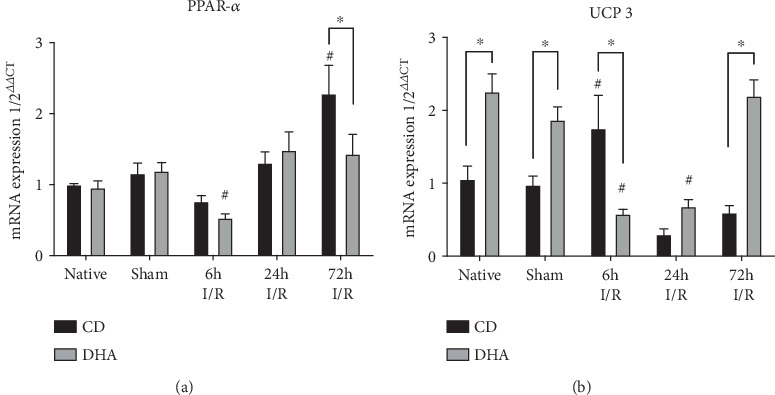
DHA pretreatment attenuates PPAR expression and enhances uncoupling protein expression post-MI; both are cardioprotective. (a) Peroxisome proliferator-activated receptor alpha (PPAR-*α*) and (b) mitochondrial uncoupling protein 3 (UCP 3) mRNA expressions were analyzed in the murine myocardium of native, sham, and MI-exposed mice that were CD or DHA supplemented. *n* = 7 mice per group, ^∗^*P* < 0.05.

Peroxisome proliferator-activated receptor alpha (PPAR-*α*) mRNA expression was lower in DHA-supplemented mice 6 h after MI compared to respective sham. In opposition, PPAR-*α* mRNA expression increased in CD mice 72 h after MI compared to DHA-supplemented and respective sham mice (Figure 6(a)). Mitochondrial uncoupling protein 3 (UCP 3) mRNA expression was increased in DHA-supplemented native and sham compared to CD mice. However, UCP 3 mRNA expression decreased in DHA supplemented, while increased in CD mice 6 h after MI compared to respective sham and DHA-supplemented mice. 24 h after MI UCP 3 mRNA expression was still reduced in DHA-supplemented mice, while returned to sham in CD groups with no difference between CD- and DHA-supplemented mice at this time point. However, UCP 3 mRNA expression increased in DHA-supplemented mice, while remained decreased in CD with a similar expression pattern compared to respective sham 72 h after MI (Figure 6(b)).

## 4. Discussion

The present study shows for the first time that modulation of the inflammatory response and adapted energy metabolism may play a role in the beneficial effect of DHA pretreatment on reperfused MI. Various studies investigating *ω*-3 FA effects on cardiac health report positive effects using either DHA, EPA, or combinations since DHA is retroconverted to EPA, especially at higher concentrations as used in our investigation [22, 24, 39, 40]. The present study used diets with similar omega-6 FA content and higher preformed DHA, but not EPA in DHA diet, in order to decrease confounding by individual and rate-limiting differences in long-chain polyunsaturated fatty acid metabolism [41]. Furthermore, the herein used concentration has been shown to increase DHA serum and tissue concentrations [31, 42], which was beneficial in pulmonary and neurological diseases in rodents [32, 33]. Male mice were chosen to eliminate the complex cardioprotective effects that have been reported for all estrogen receptor subtypes against I/R injury, and a purified diet was chosen as the basis for both diets to avoid variability in standard chows [43].

The observed LV dysfunction in CD mice after reperfused MI is in accordance with previous studies from our groups and others [44–48]. However, in the present study, DHA supplementation beginning 7 days prior to MI resulted in sustained systolic and diastolic LV function that was characterized by lower end-diastolic pressure (EDP), greater ejection fraction (EF), and reduced isovolumetric relaxation (Tau) 14 d after 60 min LAD occlusion compared to CD-fed mice, with no difference in end-systolic blood pressures. These data suggest that DHA pretreatment has physiological consequences on the development of MI-induced LV dysfunction, preserving cardiac function in mice. The observed DHA-induced attenuation of MI-induced LV dysfunction adds to cardioprotective effects of other omega-3 FA in a Langendorff perfusion model reporting that an intravenous bolus of EPA: DHA 6: 1 protects against myocardial ischemia-reperfusion-induced injury [23]. Furthermore, preserved LV function in DHA-pretreated mice 14 days after MI is in line with the clinical data from the OMEGA-REMODEL trial, demonstrating that in humans, the combination of DHA and EPA *ω*-3 FA ethyl ester supplementation beginning after the onset of STEMI improved LV function after 6 months. In summary, the preserved LV function in DHA-pretreated mice after reperfused MI is corroborated by the current clinical and experimental literature and suggests that DHA pretreatment attenuates I/R injury-induced cardiac dysfunction in mice. Our study goes beyond this to investigate molecular mechanisms of omega-3 FA-induced cardioprotection following ischemia/reperfusion injury.

Even though *ω*-3 FA effects have been heavily studied in the context of MI, little is known about the beneficial metabolic and anti-inflammatory mechanisms that decrease the risk of heart failure associated with worse prognosis after MI if unrestricted. Various studies show the therapeutic potential of attenuating cardiac disease development via modulating the inflammatory response to various insults [8, 11, 48–50].

In our study, DHA pretreatment was associated with an attenuation of cytokine expression. Furthermore, DHA leads to earlier and stronger initiation, but prompter termination of macrophage chemoattractant CCL2 expression. This data suggests that DHA treatment results in restricted inflammation, potentially attenuating remodeling in reperfused MI.

This inflammatory stimulus is also followed by an increased macrophage infiltration in DHA-treated mice 7 d after MI, returning to baseline levels after 14 d. This strong but timely restricted inflammation in DHA-treated mice corroborates our hypothesis of timely and therefore attenuated remodeling. Accordingly, DHA treatment leads to less collagen deposition, resulting in smaller infarct sizes after reperfused MI. In this regard, we found that the peak of macrophages in DHA mice after 7 d consisted mainly of alternatively activated Ly6C-positive macrophage phenotype, being accompanied by less collagen deposition and better LV function in DHA-pretreated mice after 14 d. In summary, this data suggests that a stronger remodeling stimulus leads to a more rapid and compacted scar formation and thus smaller infarct size in DHA-supplemented mice. Therefore, the beneficial effect of DHA may depend on modulation of the inflammatory response initiated by MI.

The expression of the myosin heavy chain (MHC) subunits is developmentally regulated and inappropriate expression associated with cardiomyopathies [51, 52]. Furthermore, heart failure is characterized by numerous molecular changes in contractile pathways including a switch from *α*- to *β*-MHC isoform [52–54]. *β*-MHC is characterized by lower ATP consumption and therefore higher efficiency, potentially being advantageous and implying that the *α*- to *β*-MHC shift may be an adaptive response to myocardial injury [44, 48].

In our study, *α*-MHC mRNA expression showed a transient decrease after MI in both CD- and DHA-pretreated mice, indicative of favorable molecular changes in both groups (Figure 5(a)). Further, a significant increase in *β*-MHC expression was seen in both CD- and DHA-supplemented mice after 24 h. Interestingly, *β*-MHC expression was significantly greater in CD compared to DHA-pretreated mice 24 h after MI, indicative of a lesser need for cardiomyocyte adaptation after DHA treatment (Figure 5(b)). Thus, the lower *β*-MHC mRNA upregulation in DHA-pretreated mice may be indicative of a cardioprotective mechanism possibly mediated through improved energy supply or altered metabolism.

Another important mechanism protecting cardiomyocyte adaption against myocardial injury consists of the reduction of oxidative stress, which modulates inflammatory response [55, 56]. Glutathione peroxidase (GPx) reduces peroxides to nonreactive products and decreases function that induces cardiac matrix remodeling [57]. Also, HOX-1 induction is absent in mice supplemented with DHA in strong contrast to CD; here, significant induction of HOX-1 72 hrs after MI strongly indicates antioxidative mechanisms playing a key role in DHA-mediated cardioprotection, as reported by others [44]. Therefore, our data show that DHA pretreatment reduced MI-induced GPx1- and HOX-1 expressions (Figures 5(c) and 5(d)), suggesting that DHA pretreatment induces other antioxidative mechanism potentially protecting the heart from I/R injury-induced LV remodeling.

As to this, cardiomyocytes also protect themselves against oxidative stress via mitochondrial uncoupling protein (UCP) 3, separating oxidative phosphorylation from ATP synthesis and protecting mitochondria from ROS generation [58]. Supplementation of omega-3 fatty acids specifically induces cardiac UCP 3 expression, and overexpression protects cardiomyocytes through reduced ROS generation and apoptosis from I/R injury cardiomyocyte dysfunction and preventing cardiomyocyte death, all involved in the development of LV dysfunction [59–62]. The induced UCP 3 mRNA expression seen in the myocardium of DHA supplementation sham mice is congruent with *in vitro* and *in vivo* studies potentially preventing MI-induced oxidative stress [63–65]. The fact that UCP 3 expression decreases after I/R injury in DHA-pretreated mice is surprising, but downregulation of UCP 3 has been reported after I/R injury through the nuclear transcription factor peroxisome proliferator-activated receptor- (PPAR-) *α* [66, 67]. We have previously shown that pressure overload-induced hypertrophy as well as repetitive I/R is associated with a transient downregulation of PPAR-*α* and that pharmacological reactivation of PPAR-*α* as well as MHC-specific PPAR-*α* overexpression worsens contractile function, suggesting that substrate switching from fatty acid to glucose utilization in the stressed heart may preserve contractile function [58, 68, 69]. Accordingly, we show here a transient reduction of PPAR-*α* expression in both CD and DHA mice after 6 h, but only being significant in DHA-supplemented mice compared to respective sham. This data is in accordance with previous results from the above-mentioned studies but also suggests a cardioprotective effect of DHA via reduced fatty acid uptake and oxidation. We hypothesize that significant I/R injury-induced PPAR-*α* downregulation in DHA-supplemented mice results in reduced fatty acid uptake and oxidation. We further speculate that reduced *β*-oxidation in DHA-supplemented mice may not act as an adequate trigger for early UCP 3 upregulation at this time point. Also, within time, UCP 3 induction normalizes to sham levels in DHA-supplemented mice, potentially interconnecting transient UCP 3 downregulation to PPAR-*α* expression.

In summary, our data suggest that DHA supplementation induces cardioprotection from myocardial ischemia and reperfusion injury through modulation of inflammatory response with early macrophage attraction but timely and attenuated remodeling. DHA seems to induce cardiomyocyte protection at least in part by transient PPAR-*α* downregulation with subsequently reduced UCP 3 expression and oxidation, diminishing the need for antioxidant mechanisms including mitochondrial function, or switch of MHC-isoforms from high ATP consuming *α*- to energetically more efficient *β*-isoforms.

## 5. Conclusions

Cardiac dysfunction and detrimental myocardial remodeling after MI are consequences of ROS generation, inflammation, and altered energy metabolism. Various promising experimental cardioprotective interventions have targeted individual contributors of I/R injury in animal models but have failed after translating into the clinical setting. However, MI is multifaceted and includes inflammation, ROS generation, and altered energy metabolism. Our study shows for the first time that DHA interacts with inflammation, ROS generation, LV matrix remodeling, cardiomyocyte metabolism, and contractile elements and is therefore targeting multiple mechanisms involved in cardiac I/R injury, attenuating MI-induced development of cardiac dysfunction.

## Figures and Tables

**Table 1:** Diet composition.

PUFA	Control diet	DHA diet
Linoleic (18 : 2)	3.00 g/kg	2.9 g/kg
Linolenic (18 : 3)	0.7 g/kg	0.43 g/kg
DHA (22 : 6)	0.0 g/kg	0.26 g/kg

## Data Availability

Data will made available upon request.
